# Epigenetic Control of Muscle Stem Cells: Focus on Histone Lysine Demethylases

**DOI:** 10.3389/fcell.2022.917771

**Published:** 2022-05-20

**Authors:** Delia Cicciarello, Laurent Schaeffer, Isabella Scionti

**Affiliations:** Pathophysiologie et Génétique du Neurone et du Muscle (PGNM), Institut NeuroMyoGène, Faculté de Médecine Rockefeller, Université Claude Bernard Lyon I, Villeurbanne, France

**Keywords:** histone demethylase, muscle stem cell (MuSC), cell fate and differentiation, epigenetics, metabolism

## Abstract

Adult skeletal muscle is mainly composed of post-mitotic, multinucleated muscle fibers. Upon injury, it has the unique ability to regenerate thanks to the activation of a subset of quiescent muscle stem cells (MuSCs). Activated MuSCs either differentiate to repair muscle, or self-renew to maintain the pool of MuSC. MuSC fate determination is regulated by an intricate network of intrinsic and extrinsic factors that control the expression of specific subsets of genes. Among these, the myogenic regulatory factors (MRFs) are key for muscle development, cell identity and regeneration. More globally, cell fate determination involves important changes in the epigenetic landscape of the genome. Such epigenetic changes, which include DNA methylation and post-translational modifications of histone proteins, are able to alter chromatin organization by controlling the accessibility of specific gene loci for the transcriptional machinery. Among the numerous epigenetic modifications of chromatin, extensive studies have pointed out the key role of histone methylation in cell fate control. Particularly, since the discovery of the first histone demethylase in 2004, the role of histone demethylation in the regulation of skeletal muscle differentiation and muscle stem cell fate has emerged to be essential. In this review, we highlight the current knowledge regarding the role of histone demethylases in the regulation of muscle stem cell fate choice.

## Introduction

Skeletal muscle contributes to approximatively 40% of the body weight and is thus the largest organ of the human body. Its unique regenerative capacity relies on specific muscle stem cells (MuSCs). Under physiological conditions, adult MuSCs are quiescent, reside at the periphery of the muscle fiber, between the sarcolemma and the basal lamina, and are characterized by the specific expression of the homeobox transcription factor PAX7 (Seale et al., 2000; Relaix et al., 2006). These MuSCs have the same embryonic origin than muscle precursors that form skeletal muscle during development. Upon activation (during development or by muscle injury in adults), MuSCs undergo differentiation steps involving the same set of transcription factors than embryonic muscle precursors, suggesting the same epigenetic regulations of gene transcription. MuSCs differentiation consists of a transcriptional cascade characterized by specific expression of muscle specific regulatory (MRF) genes, such as *Myf5*, *Mrf4*, *MyoD*, and *Myogenin* (Edmondson and Olson, 1989; Braun et al., 1990; Braun et al., 1994; Singh and Dilworth, 2013). Among them, MYOD is a key regulator of cell fate commitment. Conversely to the abundant knowledge accumulated on how MYOD affects chromatin organization at its target genes’ promoters during muscle differentiation (Bergstrom et al., 2002; de la Serna et al., 2005; Tapscott, 2005), the early epigenetic events involved in the activation of *MyoD* expression still lack in depth understanding. So far most of the studies concerning the regulation of *MyoD* gene expression come from the embryonic development (Asakura et al., 1995). Three regulatory regions have been identified, the Core Enhancer (CE), the distal regulatory region (DRR) and the promoter region (Goldhamer et al., 1995; Chen et al., 2001). The CE and the DRR are transcribed to produce two enhancer RNAs (CEeRNA and DRReRNA/MUNC, respectively) (Mousavi et al., 2013; Mueller et al., 2015), however, while the DRReRNA has been shown to support the activity of MYOD on its target genes, the CEeRNA is essential for the timely expression of *MyoD* and thus for the commitment of MuSCs into the differentiation process (Mousavi et al., 2013; Scionti et al., 2017). The study of the mechanisms controlling the timing of MRF expression have attracted considerable attention to the interactions between transcriptional and chromatin-remodeling factors, particularly histone modifiers. Epigenetic modifiers dynamically organize chromatin, switching its conformation from repressed to active and vice versa. They are logically essential for the control of commitment and differentiation in the muscle lineage. Among epigenetic regulators, the enzymes performing histone lysine demethylation have particularly emerged as key regulators of cell fate choice. For their activity, histone lysine demethylases (KDMs) require specific metabolic co-factors which are produced by glycolysis, mitochondrial oxidative processes or riboflavin metabolism (McCormick, 1989; Giancaspero et al., 2013; Judge and Dodd, 2020). Indeed, KDMs have been subdivided in two families depending on the metabolites they use to catalyze the demethylation of their protein targets: 1) the flavin adenine dinucleotide (FAD)-dependent family and 2) the Jumonji C (JmjC) domain-containing (JMJD) demethylases, which require α ketoglutarate (αKG) and Fe2+. These findings have pointed out the intricate and essential relationship between cell fate, transcriptional regulation and cellular metabolism. Consistently, quiescent MuSCs have a slow metabolic rate and mainly rely on fatty acid oxidation (FAO) (Fukada et al., 2007; Ito and Suda, 2014; Ryall et al., 2015). As soon as they activate, MuSCs immediate energy requirement increases and they undergo a metabolic switch from oxidative to glycolytic (Ryall et al., 2015; Yucel et al., 2019). Consistently, activated MuSCs show increased expression of genes involved in the glycolysis and tricarboxylic acid (TCA) cycle (Dell’Orso et al., 2019). Moreover, activated MuSCs possess higher mitochondrial activity than quiescent MuSCs, suggesting that cellular metabolism is not only responsible to provide energy but also to produce metabolic co-factors such as those required for KDMs (or other epigenetic modifiers) to regulate MuSC fate decisions (proliferation, commitment, fusion, and self-renewal).

In the present review, we will describe and discuss the studies linked to the role of lysine demethylases (KDM) during MuSC fate choice.

## The Role of KDM1 Family During Myogenesis and Skeletal Muscle Regeneration

Until 2004, histone methylation in eukaryotic cells was thought to be irreversible (Shi et al., 2004). The identification of the first histone demethylase in 2004, called Lysine-(k) Specific Demethylase 1 (LSD1/KDM1A), radically changed this view (Shi et al., 2004). The KDM1 demethylase family comprises LSD1/KDM1A and LSD2/KDM1B. These KDM1s are able to demethylate H3K4me1/me2 and H3K9me1/me2 through a FAD-dependent amine oxidase reaction (Shi et al., 2004). LSD1/KDM1A and LSD2/KDM1B are characterized by two specific domains: the SWIRM domain located directly downstream the N-terminal portion of the protein and the C-terminal amine oxidase like (AOL) domain, which carries the catalytic activity (Shi et al., 2004). In addition, LSD1 contains a TOWER domain that allows binding to the corepressor CoREST. LSD2 does not contain a TOWER domain but harbors CW-type and C4H2C2-type zinc finger motifs in its N-terminal part (Shi et al., 2004). This supports the hypothesis that LSD1 and LSD2 are part of different transcriptional complexes. Conversely to LSD2 (Burg et al., 2015), LSD1 has been extensively investigated in a wide variety of biological processes (Martinez-Gamero et al., 2021).

### LSD1 Demethylase Orchestrates Muscle Stem Cells Metabolic Reprogramming

**FIGURE 1:**
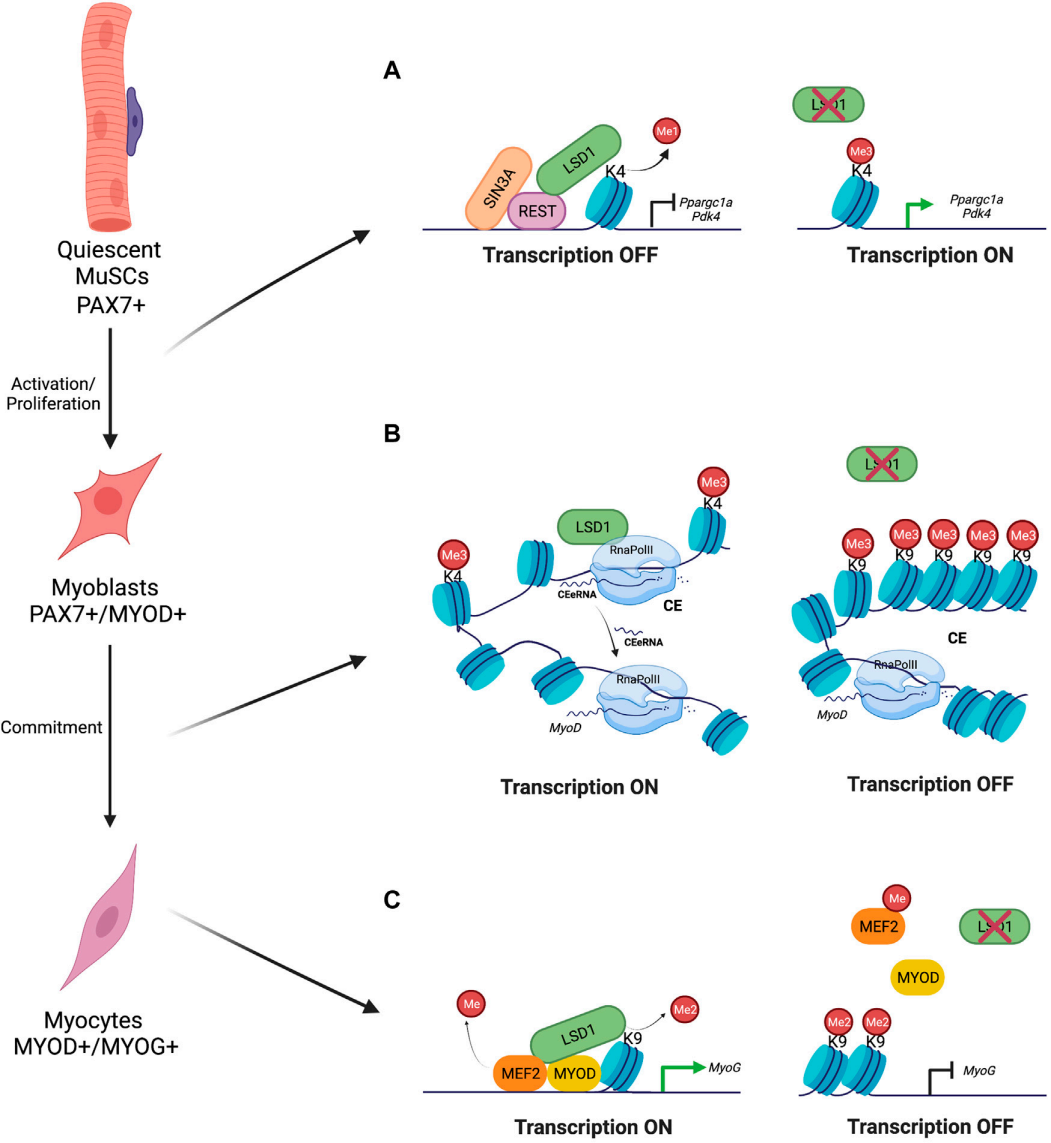
LSD1 Demethylase Regulation of Myogenesis. **(A)** During MuSCs activation and proliferation, LSD1 is enriched on promoters of oxidative metabolism genes, together with repressive factor Sin3A and REST. Here, it removes H3K4me2 marks resulting in transcription repression. LSD1 absence results in activation of oxidative metabolism genes transcription (Anan et al., 2018). **(B)** During myoblast commitment, LSD1 demethylase is recruited on MyoD core enhancer (CE), where it promotes the transcription of the CEeRNA. LSD1 ablation results in H3K9me3 methyl marks accumulation and repression of the CEeRNA transcription (Scionti et al., 2017). **(C)** During skeletal muscle differentiation, LSD1 associates with MYOD and MEF2 on Myogenin promoter. LSD1 directly demethylates MEF2D protein and removes H3K9me2 marks promoting MyoG expression. LSD1 depletion results in H3K9me2 accumulation thus inhibition of MyoG transcription (Choi et al., 2010; Choi et al., 2014).

As mentioned before, MuSCs, as other stem cells (Li et al., 2013), change their metabolic status according to their fate (Dell’Orso et al., 2019). Chromatin immunoprecipitation (ChIP)-seq analysis performed in differentiating myoblasts have shown that LSD1 is enriched at the promoter regions of genes involved in Fatty Acid oxidation (FAO) including *Ppargc1a* and *Pdk4* (Anan et al., 2018) and of genes involved in glycolytic metabolism, such as *Gapdh* (Anan et al., 2018), suggesting that LSD1 is involved in the regulation of the energy metabolism (Figure 1A). However, LSD1 Knock Down (KD) or pharmacological inhibition in differentiating myoblasts strongly increases the oxidative metabolism but has no impact on glycolytic activities. Mechanistically, LSD1 is bound to the promoter of oxidative metabolism genes, together with repressive cofactors such as Sin3A and REST (Anan et al., 2018). LSD1 inhibition causes a reduction in the level of H3K4me1 and an increase in H3K4me3 and RNApolII recruitment (Anan et al., 2018) (Figure 1A). Moreover, LSD1 was found to be enriched at two genes promoters, specific of oxidative muscle fiber (*Myh7* and *Tnnc1*), suggesting that it can influence the metabolism of newly formed myotubes. Consistently, inhibition of LSD1 activity leads to an overexpression of slow oxidative muscle fiber associated genes and to enhanced OXPHOS capacity (Anan et al., 2018). Consistently, while under glucose deprivation myotubes are less efficient in producing ATP, LSD1-inhibition confers them the ability to maintain normal ATP production *via* lipid oxidation (Anan et al., 2018).

Interestingly the LSD1 metabolic co-factor FAD is synthetized from the vitamin riboflavin (vitamin B2), thus suggesting LSD1 as the initiator of the metabolic reprogramming during myoblast differentiation (Table 1).

**TABLE 1:** List of histone demethylases that regulate MuSC fate choice.

KDMs	Substrates	Function	References
KDM1A/LSD1	H3K4me1	Activation	Ambrosio et al. (2013), Anan et al. (2018), Choi et al. (2014), Choi et al. (2010), Dentice et al. (2010), Scionti et al. (2017)
	ND	Commitment	
	H3K9me2, MEF2D	Differentiation	
KDM2B/JHDM1B	SRF	Differentiation	Kwon et al. (2021)
KDM3A/JMJD1A	H3K9me3	Activation	Liu et al. (2021)
KDM3C/JMJD1C	H3K9me2	Differentiation	Luo et al. (2017)
KDM3D/Hairless	ND	Quiescence	Liu et al. (2021)
KDM4A/JMJD2A	H3K9me2, H3K9me3	Proliferation Differentiation	Verrier et al. (2011), Zhu et al. (2021)
KDM4B/JMJD2B	H3K9me3	Differentiation	Choi et al. (2015)
KDM4C/JMJD2C	H3K9me3, MYOD	Differentiation	Jung et al. (2015)
KDM6A/UTX	H3K27me3	Differentiation	Chakroun et al. (2015), Faralli et al. (2016), Seenundun et al. (2010), Wang et al. (2013)
KDM6B/JMJD3	H3K27me3	Differentiation	Akiyama et al. (2016), McKee et al. (2022)

### LSD1 Demethylase Regulates Several Steps of Myogenesis

During myoblast differentiation *in vitro*, LSD1 protein levels increase (not the mRNA level) concomitantly to the increase of MYOD and MYOG expression (Scionti et al., 2017). Both *in vitro* and *in vivo,* the loss of LSD1 or the pharmacological inhibition of its enzymatic activity leads to a decrease of the CEeRNA expression*. In vivo* this delays *MyoD* expression in the forelimbs during embryogenesis, thus phenocopying the deletion of the CE region in the *MyoD* promoter (Chen et al., 2001; Scionti et al., 2017).

Mechanistically, LSD1 is recruited on the CE region where it activates the expression of the CEeRNA allowing timely activation of *MyoD* expression (Figure 1B). Interestingly, while the LSD1 enzymatic activity is necessary to activate the CE region, the histone modifications occurring on the CE region cannot be attributed to LSD1 (Scionti et al., 2017). Indeed, LSD1 cannot demethylate tri-methylated lysines but the absence of LSD1 induces a strong increase in histone H3K9me3 mark. Such increase could be due to the fact that LSD1 prevents H3K9me3 by removing mono- and di-methylation, or/and that LSD1 inhibition prevents the recruitment/activity of a JMJD demethylase on the CE region. Another report shows that LSD1 can indirectly regulate *MyoD* expression *via* the activation of the expression of *Dio2* (Ambrosio et al., 2013). The *Dio2* gene encodes the iodothyronine deiodinase D2, which is responsible for the conversion of the T4 prohormone to the active T3 hormone. The fine regulation of this conversion is essential to ensure the balance between proliferation and differentiation of several cell types (Dentice et al., 2010). In particular, *MyoD* promoter contains a highly conserved thyroid hormone responsive element (TREs). Moreover, LSD1 interacts with FoxO3 on the *Dio2* promoter, where it removes the repressive mark H3K9me2 to promote the expression of *Dio2* and thus of *MyoD* (Dentice et al., 2010; Ambrosio et al., 2013). Forced expression of the CEeRNA or of *MyoD* in LSD1 KD differentiating myoblasts shows that while *MyoD* expression was rescued, *Myogenin* expression was only partly restored. Consistently, the myotubes that were formed were thinner and contained less nuclei compared to controls (Scionti et al., 2017). This suggests that LSD1 is also involved in the control of *Myogenin* expression. Indeed, LSD1 was shown to directly regulate *Myogenin* expression by demethylating lysine 267 of MEF2D, thus enhancing its transcriptional activity on the *Myogenin* promoter (Choi et al., 2014). In the absence of LSD1, MEF2D remained methylated and was less recruited on the *Myogenin* promoter, which also remained enriched in the repressive mark H3K9me2 (Choi et al., 2010). These findings suggest that LSD1 regulates *Myogenin* expression by demethylating both MEF2D and H3K9me2 (Choi et al., 2010; Choi et al., 2014) (Table 1, Figure 1C).

## JMJD: Jumonji C—Domain Demethylases and Skeletal Muscle Regeneration

The JmjC demethylase family comprises members that share a JmjC domain which reacts with α-KG in an Fe^2+^_-_dependent manner (Cloos et al., 2006; Fodor et al., 2006; Klose et al., 2006b; Tsukada et al., 2006; Whetstine et al., 2006; Yamane et al., 2006). However, 2not all have been shown to be catalytically active.

### KDM2 Subfamily

**FIGURE 2:**
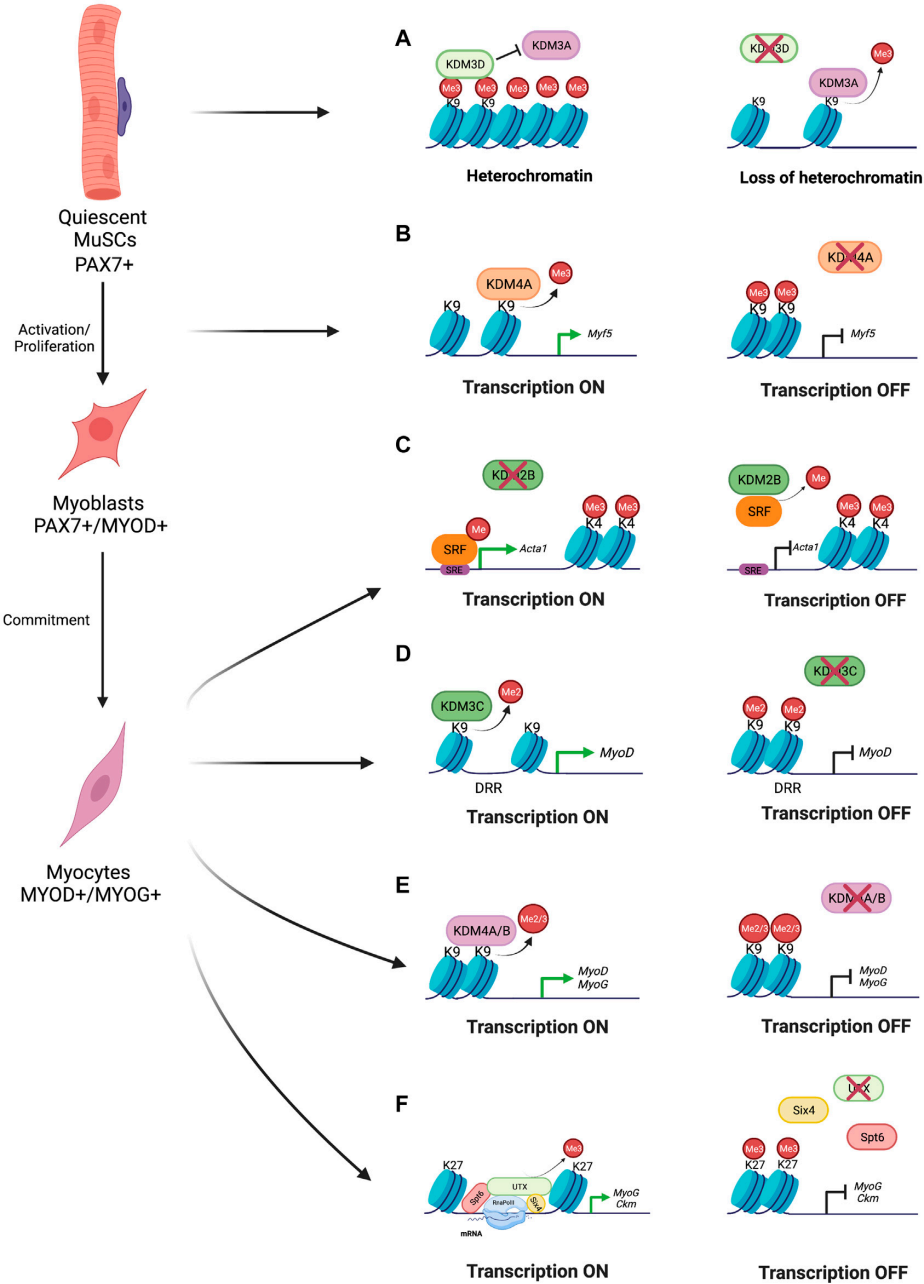
Jumonji C-domain Demethylases Role during Skeletal Muscle Regeneration. **(A)** In order to maintain MuSCs quiescence, KDM3D inhibits KDM3A demethylase activity guarantying heterochromatin integrity. In absence of KDM3D, KDM3A demethylases H3K9me3 resulting in loss of heterochromatin content (Liu et al., 2021). **(B)** Upon MuSCs activation and proliferation, KDM4A is enriched on Myf5 promoter in proliferating myoblasts, where it removes H3K9me3 histone mark allowing the transcription of Myf5 and promoting cell proliferation. Conversely, KDM4A ablation results in H3K9me3 accumulation repressing the transcription (Zhu et al., 2021). **(C)** KDM2B acts on Acta1 promoter demethylating serum response factor (SRF) and preventing its binding at serum regulatory elements (SRE), thus leading to skeletal muscle differentiation impairment. On the contrary, KDM2B absence allows SFR to bind SRE thus resulting in Acta one transcription (Kwon et al., 2021). **(D)** KDM3C demethylates H3K9me2 on the MyoD distal regulatory region (DRR). When KDM3C is depleted, MyoD transcription is inhibited and H3K9me2 marks are accumulated on DRR region (Luo et al., 2017). **(E)** During skeletal muscle differentiation, KDM4A/B remove both H3K9me2 and H3K9me3 marks on MyoD and MyoG promoters to ensure their transcription. Thus, inhibition of KDM4A/B results into inhibition of MyoD and MyoG transcription thus skeletal muscle differentiation impairment (Verrier et al., 2011; Choi et al., 2015; Zhu et al., 2021). **(F)** SIX4 transcriptional factor recruits UTX on Myogenin and Ckm regulatory regions, here, the histone chaperone SPT6 tethers UTX to the elongated RNApolII, in order to promote H3K27me3 demethylation all along the gene sequences. Conversely, absence of UTX leads to accumulation of H3K27me3 marks thus resulting in transcription repression (Seenundun et al., 2010; Wang et al., 2013; Chakroun et al., 2015).

The KDM2 subgroup consists of two members: KDM2A and KDM2B. KDM2 proteins demethylate the active chromatin marks H3K4me3 and H3K36me1/me2 and thereby act as a transcriptional co-repressor. In addition to the JmjC domain, this subgroup is characterized by several distinct domains: 1) the CXXC zinc finger DNA binding domain; 2) the PHD and the F-box domain; 3) leucine-rich repeats (Klose et al., 2006a). Mis-regulation of KDM2 family members has been observed in different pathological conditions and were thus widely investigated. However, their physiological role in normal condition is still poorly understood. So far, only the role of KDM2B have been studied in skeletal muscle, although KDM2B its absent in adult skeletal muscle (Kwon et al., 2021). KDM2B is highly expressed in muscle during late embryonic development and constantly decreases in skeletal muscle tissue with the age, supporting the idea that KDM2B needs to be silenced to achieve muscle differentiation. Consistent, overexpression of KDM2B, in cultured myoblasts, inhibits the expression of *MyoD* and *Myogenin*, and consequently inhibits differentiation. Interestingly, ChIP experiments performed in myoblasts overexpressing KDM2B, revealed that H3K4me3 levels were not altered on muscle specific promoters. Mechanistically, KDM2B seems to act through the serum response factor (SRF) since it was shown to demethylate SRF, thus preventing its binding to the serum response elements (SRE) present in the promoters of muscle specific genes (Kwon et al., 2021) (Table 1, Figure 2C).

### KDM3 Subfamily

The KDM3 subfamily is composed of four members: KDM3A/JMJD1A, KDM3B/JMJD1B, KDM3C/JMJD1C, and KDM3D/Hairless. KDM3 proteins possess a JmjC domain at the C-terminus and two non-catalytic motifs (C_2_HC_2_) zinc (Zn) finger, and LXXLL for interacting with nuclear hormone receptors (NRs). KDM3D also carries a nuclear matrix targeting signal, a nuclear localization signal and two ϕXXϕϕ motifs that participate to the binding to thyroid hormone receptors (Pilka et al., 2015). While the demethylase activity of KDM3C remains to be confirmed (Kooistra and Helin, 2012; Brauchle et al., 2013), the other three members are able to demethylate H3K9me1/me2 (Brauchle et al., 2013). It has been widely shown that KDM3 proteins have a critical role in many physiological and pathological processes, especially in cancer (Yoo et al., 2020). However, their role during myogenesis is only beginning to emerge.

**KDM3D** is expressed in quiescent adult MuSCs where it plays a critical role in the maintenance of the quiescence (Liu et al., 2021). Upon muscle injury KDM3D level decreases and it is eventually restored in self-renewed MuSCs that are back to quiescence. Inactivation of the *KDM3D* gene increases the number of activated MuSCs after muscle injury, but they die instead of participating to regeneration. The loss of MuSCs leads to skeletal muscle regeneration impairment and to a reduced number of self-renewed MuSCs (Liu et al., 2021). Interestingly, in absence of KDM3D, while the H3K9me2 level is unaffected, a massive decrease of the repressive H3K9me3 mark occurs, indicating a loss of heterochromatin (Liu et al., 2021). In addition, KDM3D Knock out (KO) MuSCs are more sensitive to genotoxic stress and DNA damage (Liu et al., 2021). Mechanistically, KDM3D binds H3K9me2/me3 in order to inhibit **KDM3A** demethylase activity and thus maintain the heterochromatin integrity in MuSC quiescence (Liu et al., 2021) (Figure 2A).

**KDM3C** plays a critical role during myoblast differentiation. The loss of KDM3C increases H3K9me2 on the *MyoD*-DRR and decreases the expression of *MyoD* and *Myogenin*, resulting in an inhibition of myoblast differentiation (Luo et al., 2017). Mechanistically, KDM3C demethylates H3K9me2 on the *MyoD*-DRR in differentiating myoblast and thus participates to the activation of the expression of *MyoD* and of *Myogenin* (Luo et al., 2017) (Figure 2D). *In vitro* analysis showed that, KDM3C activity is finely controlled by Deltex2, a Notch-binding protein, which mediates its mono-ubiquitination and promotes its degradation. Consistently, upon muscle injury, Deltex2 KO MuSCs show a dramatic increase of *MyoD* and *Myogenin* expression which leads to premature and impaired muscle regeneration. This phenotype is rescued by the downregulation of KDM3C expression (Table 1) (Luo et al., 2017).

### KDM4 Subfamily

Four demethylases, KDM4A-B-C-D-E (JMJD2A-B-C-D-E), characterize this subfamily.

Structurally, while all the KDM4 demethylases contain the JmJC catalytic domain, only the A, B, and C members possess in their C-terminal part two plant homeodomains (PHD) and two Tudor domains, which confer them important histone reader functions (Pilka et al., 2015). KDM4A and C were shown to demethylate both H3K9me2/3 and H3K36me2/3 (Labbé et al., 2013).

The expression of KDM4 family members is tightly regulated to ensure proper function in diverse biological processes, such as cellular differentiation. Perturbing their expression leads to the progression of diverse disease as cancer. In this context, recent studies have described the potential role of KDM4A-B-C during skeletal muscle development and differentiation.

KDM4 genes are differentially expressed during skeletal muscle differentiation, in particular, **KDM4A** and **KDM4B** expression steadily increases during differentiation (Choi et al., 2015). ChIP assays have shown that when myoblasts are induced to differentiate, KDM4A and KDM4B are enriched on the *MyoD* and *Myogenin* promoters, where they remove the H3K9me2/3 marks, thus participating to the activation of their expression and allowing myoblasts differentiation into myotubes (Verrier et al., 2011) (Figure 2E). While KDM4B KD only delays myoblast differentiation, KDM4A KO irreversibly impairs myogenesis (Verrier et al., 2011; Choi et al., 2015). Recently, conditional inactivation of KDM4A in MuSCs using a *Myf5*-CRE driver confirmed the essential role of KDM4A for myogenesis during embryogenesis and in adult upon muscle injury. In the absence of KDM4A, damaged muscles display less differentiating (PAX7+/MYOD+) cells and proliferating (PAX7+/Ki67+) myoblasts and regeneration is impaired (Verrier et al., 2011; Zhu et al., 2021). Mechanistically, ChIP experiments revealed that in proliferating myoblasts KDM4A is enriched on the *Myf5* promoter where it removes H3K9me3, which leads to *Cyclin D1* expression, thus promoting cell proliferation (Zhu et al., 2021) (Figure 2B).

**KDM4C** expression increases during the first phases of differentiation and decrease at later stages, when myotubes formation occur (Choi et al., 2015). In proliferative myoblasts KDM4C is both cytoplasmic and nuclear while in myotubes it localizes mostly in the nucleus (Jung et al., 2015). KDM4C interacts with MYOD and this interaction prevents MYOD methylation by the methyltransferase G9a (Jung et al., 2015). This results in MYOD stabilization by preventing its ubiquitination and degradation. Moreover, KDM4C demethylases H3K9me3 on myogenic gene promoters leading enhances transcription of myogenic specific genes, as part of the MYOD transcriptional complex (Table 1) (Jung et al., 2015).

### KDM6 Subfamily

The KDM6 family is composed of three members: KDM6A/UTX, KDM6B/JMJD3 and KDM6C/UTY. While KDM6A and KDM6B are both able to directly demethylate H3K27me2/3, there are no evidences regarding KDM6C demethylase activity (Shpargel et al., 2014). In addition to their C-terminal Jumonji C (JmjC) domain, at their N-terminus KDM6A and KDM6C contain three tetratricopeptide repeat (TPR) domains that mediate the protein-protein interaction (Pilka et al., 2015). KDM6B harbors only the JmjC domain at the C-terminal part of the protein. KDM6A and KDM6B are part of the MLL3/4 methyltransferase complex. Their role as oncogenic activators has been widely investigated in different cancer types.

The **KDM6A/UTX** gene is located on the X chromosome but escapes X inactivation in females and is ubiquitously expressed (Greenfield et al., 1998). UTX plays a crucial role during embryonic development since UTX KO embryos display reduced number of somites, failure in neural tube closure and heart defects. While knocking out UTX in female is embryonic lethal, a small percentage of UTX KO males survive and are fertile, besides their reduced size (Shpargel et al., 2014). The UTX first contribution to adult myogenesis was investigated in 2010 (Seenundun et al., 2010). Twenty-4 h after triggering myoblast differentiation, UTX occupies the *Myogenin* promoter and the *Ckm* enhancer region and this parallels a reduction in the H3K27me3 repressive mark (Seenundun et al., 2010). Interestingly, by 48 h of differentiation UTX is enriched within the coding sequence of its target genes, leading to a massive loss of H3K27me3 and allowing high expression of both *Myogenin* and *Ckm* (Seenundun et al., 2010). Upon muscle injury, the *UTX* gene inactivation or inhibition of UTX enzymatic activity in MuSCs impairs regeneration: less and smaller myofibers are formed and regenerating muscles contain more necrotic tissue and inflammatory cell infiltration (Faralli et al., 2016). In the absence of UTX, quiescence, activation and proliferation of adult MuSCs are not affected, but upon activation, the cells are unable to exit the cell cycle and remain proliferative, preventing differentiation. Mechanistically, the transcription factor SIX4 is responsible for the initial recruitment of UTX on *Myogenin* and *Ckm* regulatory regions (Chakroun et al., 2015), where the histone chaperone SPT6 tethers UTX to elongating RNApolII to allow the removal of the H3K27me3 mark all along the coding sequences of these genes (Figure 2F). Consistently, pharmacological inhibition of the transcriptional elongation by RNApolII (Seenundun et al., 2010), *Six4* KD (Chakroun et al., 2015) or *Spt6* KD (Wang et al., 2013) decrease enrichment of UTX on both *Myogenin* and *Ckm* genomic loci, leading to an increase of the H3K27me3 heterochromatin mark.

Compared to UTX, **KDM6B** seems to have a more global action in the genome (McKee et al., 2022). Ectopic expression of the catalytic domain of KDM6B in human pluripotent stem cells (hPSC) induces a massive genome wide reduction of H3K27me3, promoting MYOD-mediated myogenic differentiation of hPSC (Akiyama et al., 2016). Conversely, KDM6B KD strongly increases H3K27me3 levels and reduces the expression of both muscle specific genes and Wnt-family genes (Table 1) (McKee et al., 2022).

## Conclusion

Epigenetic control of gene expression plays a central role in MuSCs during embryonic and adult myogenesis. While the role of histone lysine demethylases is widely investigated in cancer field, their implication in the regulation of skeletal muscle differentiation is less advanced. Our current knowledge on the role of histone demethylases in the regulation of myogenesis indicates that the majority of KDMs are enriched on the promoters and regulatory regions of MRF genes, where they are required to activate gene expression and thus promote myogenesis. KDMs, as other enzymes, need metabolic co-factors to demethylate their target proteins, thus linking the epigenetic control of gene expression to MuSC metabolism. Interestingly, all the studies, described so far, have shown the crucial role of KDMs upon MuSC activation, when the metabolic reprogramming is already achieved. However, little is known about the early metabolic changes during MuSC fate choice. This is mostly associated with the dissociation and purification protocols of MuSCs, which do not guarantee the quiescent state of these cells. The constant improvement of isolation protocols and techniques and the refinement of analytical methods which require smaller number of cells, will help to decipher the relationship between KDMs, cell metabolism and gene expression during the quiescent stage. This will provide crucial information to understand the epigenetic regulation of skeletal muscle development and regeneration, and will help to elucidate the molecular pathways involved in the different steps of the myogenic program, from muscle progenitors to mature myofibers, a central challenge in regenerative medicine.
